# Variation in the provision and practice of implant-based breast reconstruction in the UK: Results from the iBRA national practice questionnaire

**DOI:** 10.1016/j.breast.2017.07.016

**Published:** 2017-10

**Authors:** Senthurun Mylvaganam, Elizabeth Conroy, Paula R. Williamson, Nicola L.P. Barnes, Ramsey I. Cutress, Matthew D. Gardiner, Abhilash Jain, Joanna M. Skillman, Steven Thrush, Lisa J. Whisker, Jane M. Blazeby, Shelley Potter, Christopher Holcombe, iBRA Steering Group, N.L.P. Barnes, J.M. Blazeby, O.A. Branford, E.J. Conroy, R.I. Cutress, M.D. Gardiner, C. Holcombe, A. Jain, K. McEvoy, N. Mills, S. Mylvaganam, S. Potter, J.M. Skillman, E.M. Teasdale, S. Thrush, L.J. Whisker, P.R. Williamson, the Breast Reconstruction Research Collaborative, L. Tang, D. Nguyen, R. Johnson, V. Muralikrishnan, S. Chopra, A. Reid, S. Benyon, C. Murphy, F. Soliman, V. Lefemine, S. Saha, K. Ogedegbe, O.S. Olyinka, J.R. Dicks, N. Manoloudakis, F. Conroy, G. Irwin, S. McIntosh, I. Michalakis, S. Hignett, R. Linforth, R. Rathinaezhil, H. Osman, K. Anesti, M. Griffiths, R. Jacklin, A. Waterworth, R. Foulkes, E. Davies, K. Bisarya, A. Allan, J. Leon-Villapalos, F.A.K. Mazari, I. Azmy, S. George, F.S. Fahmy, A. Hargreaves, J. Seward, S. Hignett, J. Henton, T. Collin, G. Irwin, P. Mallon, J. Turner, W. Sarakbi, I. Athanasiou, C. Rogers, M. Youssef, T. Graja, S. Huf, H. Deol, R. Brindle, S. Gawne, D. Egbeare, I. Dash, M. Galea, S. Laws, S. Tayeh, L. Parvanta, S. Down, D. Westbroek, J.W. Roberts, J. Massey, P. Turton, R. Achuthan, M. Fawzy, M. Dickson, A.R. Carmichael, A. Akingboye, R. James, K. Kirkpatrick, E. Nael, R. Vidya, S. Potter, A. Thorne, M. Rostom, I. Depasquale, S.J. Cawthorn, T. Gangamihardja, S. Joglekar, J. Smith, A. Halka, D. MacMillan, S. Clark, B. Pearce, L. Mansfield, I. King, A. Hazari, B. Smith, A.J. Volleamere, D. Egbeare, D. Ferguson, N. Barnes, C. Holcombe, A. Knight, F. MacNeill, A. Conway, T. Irvine, S. Mylavaganam, N. Dunne, S. Kohlhardt, C. Hoo, S. Kirk, J. Hu, S. Ledwidge, S. Tang, D. Banerjee, S. Waheed, V. Voynov, S. Soumian, J. Henderson, J. Harvey, S. Robertson, R.I. Cutress, S. Mylvaganam, R. Waters, A. Carbone, J. Skillman, Ansar Farooq, H. Tafazal, D. Clarke, D. Cocker, L.M. Lai, J. Winter Beatty, M. Barkeji, R. Vinayagam, K. McEvoy, M. Mullan, C. Osborne, E. Baker, J. Piper

**Affiliations:** m Abertawe Bro Morgannwg University Health Board NHS Trust, UK; n Addenbrookes, UK; o Airedale NHS Foundation Trust, UK; p Aneurin Bevan Health Board, UK; q Barking Havering and Redbridge NHS Trust, UK; r Barnsley District General Hospital, UK; s Bedford Hospital/Bedfordshire NHS Trust, UK; t Belfast Health and Social Care Trust, UK; u Blackpool Teaching Hospitals NHS Foundation Trust, UK; v Bradford Teaching Hospitals NHS Foundation Trust, UK; w Brighton and Sussex University Hospitals NHS Trust, UK; x Broomfield Hospital Mid Essex NHS Trusts, UK; y Calderdale and Huddersfield NHS Trust, UK; z Cardiff and Vale, UK; aa Chelsea and Westminster Hospital NHS Foundation Trust, UK; ab Chesterfield Royal Hospital NHS Foundation Trust, UK; ac Countess of Chester Hospital NHS Foundation Trust, UK; ad County Durham and Darlington NHS Foundation Trust, UK; ae Craigavon Hospital – Southern Health and Social Care Trust, UK; af Croydon University Hospital, UK; ag Doncaster and Bassetlaw Hospitals, UK; ah Dorset County Hospital NHS Foundation Trust, UK; ai East and North Herts NHS Trust, UK; aj East Lancashire Hospitals Trust, UK; ak Frimley Health NHS Foundation Trust, Frimley Park Hospital Site, UK; al Great Western Hospital, Swindon, UK; am Hampshire Hospitals NHS Foundation Trust, UK; an Homerton University Hospital, UK; ao James Paget University Hospital, UK; ap Kings College Hospital, UK; aq Leeds Teaching Hospital NHS Trust, UK; ar Lister Hospital, East and North Herts, UK; as London Breast Institute, UK; at Luton and Dunstable University Hospital, UK; au Mid Staffordshire NHS Foundation Trust, UK; av Musgrove Park Taunton, UK; aw NHS Grampian, UK; ax North Bristol NHS Trust, UK; ay North Middlesex University Hospital, UK; az Northern Lincolnshire and Goole Hospitals NHS Foundation Trust, UK; aaa Nottingham University Hospitals NHS Trust, UK; aab Poole Hospital NHS Foundation Trust, UK; aac Portsmouth Hospitals NHS Trust, Queen Alexandra Hospital, UK; aad Queen Victoria Hospitals NHS Foundation Trust, East Grinstead, UK; aae Royal Berkshire Hospital, UK; aaf Royal Bolton Foundation Trust, UK; aag Royal Devon and Exeter NHS Foundation Trust, UK; aah Royal Liverpool and Broadgreen, UK; aai Royal Marsden NHS Foundation Trust, UK; aaj Royal Surrey County Hospital NHS Foundation Trust, UK; aak Royal Wolverhampton Hospitals NHS Trust, New Cross Hospital, UK; aal Sheffield Teaching Hospitals, UK; aam South Eastern Trust, Northern Ireland, UK; aan St Bartholomew's Hospital, UK; aao St George's Healthcare NHS Trust, UK; aap Surrey and Sussex NHS Trust, UK; aaq University Hospitals North Staffordshire NHS Trust, UK; aar University Hospital South Manchester, UK; aas University Hospital Southampton, UK; aat University Hospitals Birmingham, UK; aau University Hospitals Coventry and Warwickshire, UK; aav Warrington & Halton Hospitals NHS Foundation Trust, UK; aaw Warwick Hospital, UK; aax West Herfordshire Hospitals NHS Trust, UK; aay West Middlesex University Hospital, UK; aaz Wirral University Teaching Hospital NHS Foundation Trust, UK; aaaa Worcestershire Acute Hospitals NHS Trust, UK; aaab Yeovil District General Hospital, UK; aaac York Hospitals NHS Foundation Trust, UK; a New Cross Hospital, Royal Wolverhampton Hospitals NHS Trust, Wednesfield Way, Wolverhampton, WV10 0QP, UK; b Clinical Trials Research Centre (CTRC), North West Hub for Trials Methodology/University of Liverpool, Liverpool, L12 2AP, UK; c Breast Unit, University Hospital of South Manchester NHS Foundation Trust, Southmoor Road, Manchester, M23 9LT, UK; d Breast Unit, University Hospital Southampton, Tremona Road, Southampton, Hampshire, SO16 6YD, UK; e Faculty of Medicine, Cancer Sciences Unit, University of Southampton, Somers Cancer Research Building, University Hospital Southampton, Tremona Road, Southampton, SO16 6YD, UK; f Nuffield Department of Orthopaedics, Rheumatology and Musculoskeletal Sciences, University of Oxford, Nuffield Orthopaedic Centre, Windmill Road, Headington, Oxford, OX3 7HE, UK; g Department of Plastic Surgery, Imperial College London NHS Trust, London, SW7 2AZ, UK; h Department of Plastic Surgery, University Hospitals Coventry and Warwickshire NHS Trust, Clifford Bridge Road, Coventry, CV2 2DX, UK; i Breast Unit, Worcester Royal Hospital, Charles Hastings Way, Worcester, WR5 1DD, UK; j Breast Institute, Nottingham University Hospitals NHS Trust, Hucknall Road, Nottingham, NG5 1PB, UK; k Bristol Centre for Surgical Research, School of Social and Community Medicine, University of Bristol, 39 Whatley Road, Clifton, Bristol, BS8 2PS, UK; l Linda McCartney Centre, Royal Liverpool and Broadgreen University Hospital, Prescot Street, Liverpool, L7 8XP, UK

**Keywords:** Survey, Implant-based reconstruction, Acellular dermal matrix, Dermal sling, Current practice, Breast reconstruction

## Abstract

**Introduction:**

The introduction of biological and synthetic meshes has revolutionised the practice of implant-based breast reconstruction (IBBR) but evidence for effectiveness is lacking. The iBRA (implant Breast Reconstruction evAluation) study is a national trainee-led project that aims to explore the practice and outcomes of IBBR to inform the design of a future trial. We report the results of the iBRA National Practice Questionnaire (NPQ) which aimed to comprehensively describe the provision and practice of IBBR across the UK.

**Methods:**

A questionnaire investigating local practice and service provision of IBBR developed by the iBRA Steering Group was completed by trainee and consultant leads at breast and plastic surgical units across the UK. Summary data for each survey item were calculated and variation between centres and overall provision of care examined.

**Results:**

81 units within 79 NHS-hospitals completed the questionnaire. Units offered a range of reconstructive techniques, with IBBR accounting for 70% (IQR:50–80%) of participating units' immediate procedures. Units on average were staffed by 2.5 breast surgeons (IQR:2.0–3.0) and 2.0 plastic surgeons (IQR:1.0–3.0) performing 35 IBBR cases per year (IQR:20-50). Variation was demonstrated in the provision of novel different techniques for IBBR especially the use of biological (n = 62) and synthetic (n = 25) meshes and in patient selection for these procedures.

**Conclusions:**

The iBRA-NPQ has demonstrated marked variation in the provision and practice of IBBR in the UK. The prospective audit phase of the iBRA study will determine the safety and effectiveness of different approaches to IBBR and allow evidence-based best practice to be explored.

## Highlights

• Implant breast reconstruction (IBBR) and the range of techniques is poorly evidence based.
• We aimed to explore the current practice of IBBR in the UK to inform the design of a future definitive study.
• Significant variation was demonstrated in the availability of techniques and patient selection for IBBR.
• There is a need for well-designed studies to establish best practice and improve outcomes for patients considering IBBR.

## 1 Introduction

Implant-based breast reconstruction (IBBR) is the most commonly-performed reconstructive procedure in the UK [1]. Traditionally, this has been a two-stage procedure in which a tissue-expander is placed under the pectoral muscle at the time of mastectomy and inflated over several weeks before being exchanged for a fixed-volume implant. This type of IBBR can produce good cosmetic results for patients with small to medium-sized breasts with minimal ptosis [2]. Patients with larger or more ptotic breasts desiring reconstruction have historically been offered autologous tissue-based techniques, often latissimus dorsi flap reconstruction with or without an implant to achieve the desired volume [3].

In the last ten years, the introduction of new techniques using biological or synthetic meshes to augment the subpectoral pocket have revolutionised the practice of IBBR. These products allow single-stage direct-to-implant reconstruction [4] avoiding the need for time-consuming and uncomfortable expansions and a second operation [5]. They have also broadened the indications for IBBR so that more women are suitable for a mesh-assisted reconstruction than the more traditional tissue-expansion-based techniques. This is because the mesh enlarges the subpectoral pocket improving lower-pole projection and creating a more natural-looking, ptotic result [6], [7], [8], [9], [10]. These techniques of IBBR therefore have significant potential benefits for patients and healthcare providers.

Despite the widespread adoption of mesh-assisted IBBR into practice, there is a lack of high-quality evidence to support the proposed benefits of these techniques [11]. Recent events leading to the temporary withdrawal of Strattice in France [12] due to excessively high rates of complications have highlighted the need for robust evaluation of mesh-assisted IBBR to protect patients and prevent potentially inferior interventions or products becoming established in practice. Randomised trials (RCTs) are needed, but to-date only five RCTs have been attempted; three comparing IBBR with and without mesh and two comparing different products. Of the three comparing IBBR with and without mesh, the first, a North-American trial, compared two-stage IBBR with and without biological mesh [13]. This recruited slowly and was closed early to accrual by the Data Safety Monitoring Board. The second [14], from the Netherlands, compared two-stage IBBR with single-stage direct-to-implant reconstruction with acellular dermal matrix (ADM). This suggested unacceptably high complication rates in the ADM group (29% vs 5%) and the trial was stopped due to safety concerns. The complication rates seen in the ADM group, however, do not reflect those seen in observational studies in the UK [15] and it has been suggested that the results may reflect the surgeons' initial learning curve rather than problems with the technique [16]. A third Canadian study also comparing standard two-stage expander-implant reconstruction and single-stage mesh-assisted IBBR is yet to report [17]. Two further studies have compared different meshes. One North American trial compared two human ADMs [18] and demonstrated no difference between products and a second trial from Europe compared biological and synthetic meshes [19]. This latter study, although reported as a pilot-study, was in fact a small trial that was insufficiently well-designed to identify differences between the treatment groups [20]. To date, data from the UK comprise heterogeneous, often single-centre cohort studies or case-series [21], [22], much of which has been reported only in abstract form [23], [24]. There is therefore now a need for a well-designed and conducted multi-centre pragmatic trial to inform UK practice.

Before such a trial can be conducted, it is necessary to define the research question and determine the optimal study design. It is therefore important to understand the current practice of IBBR. Uncertainties relating to numbers of procedures being performed; the numbers of surgeons and centres offering each technique; the products being used and selection criteria for different techniques need to be addressed. It is also vital to explore which study designs would be the most acceptable to both patients and surgeons to optimise participation and recruitment. Pilot work is required to establish these parameters before a trial can be considered.

The iBRA (implant Breast Reconstruction evAluation) study is a trainee collaborative project that aims to explore the practice and outcomes of IBBR to inform the design and conduct of a future trial [25]. We report the results of the first phase of the iBRA Study, a National Practice Questionnaire (NPQ) which aimed to survey breast and plastic surgical units across the UK to comprehensively describe the current practice of IBBR.

## 2 Methods

The national practice questionnaire (NPQ, Appendix 1) was developed in February 2014 by members of the iBRA steering group based on a comprehensive review of the literature [11], current professional guidelines [26], [27] and clinical expertise. It included sections evaluating the availability of different approaches to breast reconstruction and the numbers of surgeons performing reconstructive techniques at each unit; volumes of implant-based procedures performed; availability and use of different meshes and techniques and selection criteria for each procedure type. Respondents were also asked whether they felt their practice of IBBR had changed following the introduction of mesh-assisted techniques and if so, what the change had been. The questionnaire was piloted with surgeons at two hospitals to ensure face and content validity prior to circulating the questionnaire nationally.

All breast and plastic surgical units performing mastectomy with or without immediate breast reconstruction in the UK were eligible for inclusion. Trainees were invited to participate via the Mammary Fold breast trainees' group, the Reconstructive Surgery Trials Network (RSTN) and the National Trainee Research Collaborative (NTRC). The professional associations (Association of Breast Surgery (ABS) and British Association of Plastic, Reconstructive and Aesthetic Surgeons (BAPRAS)) endorsed the study and encouraged units to participate. Each participating trainee was required to identify a consultant lead in their unit. The trainee completed the questionnaire with this consultant ensuring that responses reflected the practice of the unit as a whole, rather than that of an individual surgeon.

Study data were collected and managed using REDCap electronic data capture tools hosted at University of Edinburgh [28].

### 2.1 Analysis

Descriptive summary statistics were calculated for each survey item to evaluate the availability, volume and indications for each type of IBBR. Categorical data was summarised by counts and percentages. Continuous data was summarised by median, interquartile range (IQR) and range. No data imputation methods were used for items with no response and when a unit did not complete a specific section of the questionnaire, it was assumed that the unit did not offer that approach. Statistical Analysis Software (SAS^®^ 9.1.3; SAS Institute Inc., Cary, NC, USA) was used for all analyses. Free text responses were collated and analysed using a content analysis approach.

## 3 Results

### 3.1 Participation

Between May 2014 and November 2015, 81 responders from 79 NHS hospitals completed the NPQ. Two hospitals had independent responses from the breast and plastic surgical units. Each of these was considered an independent unit with different practices despite stemming from the same organisation. 67 of 144 (47%) breast units and 14 of 53 (26%) plastic units in the UK participated in the study.

### 3.2 Unit demographics

Unit demographics are shown in Table 1. 79/81 (98%) units specified the types of breast reconstruction that their unit offered. All (79/79, 100%) offered patients implant-based techniques and most offered latissimus dorsi flaps (76/79, 96%) and therapeutic mammaplasty (77/79, 97%). Almost half of units provided an on-site free flap service with 43% (34/79) offering DIEP (deep inferior epigastric perforator) flaps (Table 1).

**Table 1:** Characteristics of participating units.

Unit characteristic	N = 79
**Types of breast reconstruction offered**	
Implant-based reconstruction	79 (100)
Pedicled flaps	
Latissimus dorsi	76 (96)
Pedicled TRAM	31 (39)
Free flaps	
DIEP	34 (43)
Other autologous (e.g SGAP, IGAP, TUG, SIEA)	24 (30)
Therapeutic mammoplasty	75 (95)
Revisional surgery	77 (97)
**Number of staff performing breast and reconstructive surgery**	
**Breast surgery**	
Number of consultant surgeons with an interest in breast surgery (FTE, median, IQR, range)	3.0 (2.0–3.8) (0.0–7.0)
Number of consultant breast surgeons who perform reconstructive surgery (FTE, median, IQR, range)	2.5 (2.0–3.0) (0.0–7.0)
**Plastic surgery**	
Number of consultant plastic surgeons with an interest in breast surgery (FTE, median, IQR, range)	1.0 (0–3.0) (0.0–21.0)
Number of consultant plastic surgeons who perform reconstructive surgery (FTE, median, IQR, range)	2.0 (1.0–3.0) (0.0–10.0)
**Number of immediate implant-based breast reconstructions performed per year** (median, IQR, range)	35 (20-50) (0-230)
**Percentage of immediate breast reconstructions that are implant-based**	*Med (IQR)* 70.0 (50.0–80.0)
**Approaches to implant-based reconstruction offered**	*n (n/N%), N* = *79*
Standard 2 stage submuscular placement	60 (75.9)
Reduction pattern with dermal sling	66 (83.5)
Acellular dermal matrix assisted reconstruction	59 (74.7)
Other non-dermal biological-assisted reconstruction	19 (24.1)
TiLOOP assisted reconstruction	19 (24.1)
Other synthetic assisted reconstruction	8 (10.1)
**Types of biological mesh used**	*n (n/N%),* N = 62
Strattice	53 (85.5)
SurgiMend	33 (53.2)
Veritas	8 (12.9)
XCM	5 (8.1)
Seri	4 (6.5)
BioDesign	3 (4.8)
Permicol	3 (4.8)
Native	2 (3.2)
Other	1 (1.6)
**Types of synthetic mesh used (n** = **25)**	*n (n/N%), N* = *25*
TiLOOP	20 (80.0)
TiGR	1 (4.0)

DIEP – deep inferior epigastric perforator; FTE – full time equivalent; IQR – interquartile range; TRAM – transverse rectus abdominus myocutaenous flap.

Participating units were staffed by a median of three full-time equivalent (FTE) breast surgeons (IQR 2.0–3.8), 2.5 of whom performed reconstructive surgery (IQR 2.0–3.0). Of the participating breast units, 30/67 (45%) had access to plastic surgeons within the unit (e.g. plastic surgical clinics but no on-site access to free-flap reconstruction) and 23/67 (34%) had comprehensive on-site plastic surgical services with access to free-flaps within the unit.

### 3.3 Practice of implant-based reconstruction

**Fig. 1:**
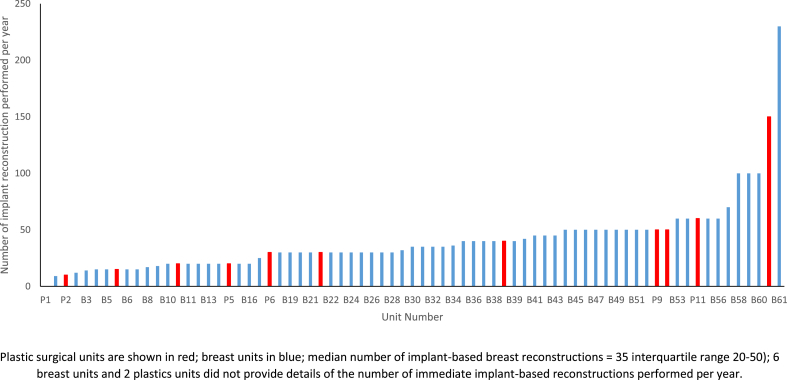
Number of immediate implant-based breast reconstructions performed per year by participating unit.

Participating units reported that IBBR represented approximately 70% (IQR 50–80%) of their reconstructive case load and performed median of 35 cases per year (IQR 20-50) (Table 1). The number of IBBR per year performed by each participating unit is shown in Fig. 1. Marked variability, however was demonstrated in the types of IBBR offered. Dermal-sling techniques were the most widely-available with over 80% (66/79, 84%) of responding units performing this technique. Three-quarters of units (59/79, 75%) offered patients acellular dermal matrix (ADM)-assisted reconstruction, but exceptional funding was required on an individual patient basis at almost 20% of centres (11/79) and less than a quarter of units (n = 19/79, 24%) performed synthetic mesh-assisted procedures. Strattice (53/61, 87%) and SurgiMend (n = 33/62, 53%) were the most commonly-used biological meshes and TiLOOP was the most frequently-used synthetic mesh, but a range of different products were reported (Table 1).

The majority of respondents reported that the introduction of the mesh-assisted procedures had changed their reconstructive practice. Almost two-thirds (48/72, 67%) felt that the ratio of immediate implant-based to autologous reconstruction had increased as a result of the new techniques largely due to increased indications for implant-based procedures and almost half (32/72, 44%) reported performing more immediate breast reconstruction since the new techniques had been introduced.

### 3.4 Patient selection for new techniques of implant-based breast reconstruction

**Fig. 2:**
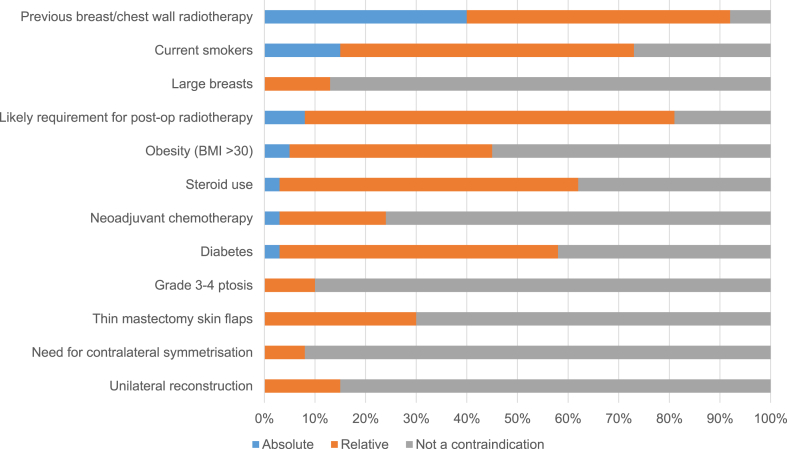
Patient selection for dermal sling procedures (n = 66).

There was significant variation in patient selection for different techniques across the responding units. The majority of units (54/66, 82%) reported offering dermal-sling techniques to women with ptotic breasts but there was a lack of consensus regarding other aspects of patient selection. Previous radiotherapy to the breast, for example was considered an absolute contraindication to the technique by almost 40% (25/66, 38%) of responding units; a relative contraindication by approximately half of units (32/66, 48%) and not a contraindication by almost 10% (5/66, 8%), demonstrating significant variation in practice (Fig. 2).

**Fig. 3:**
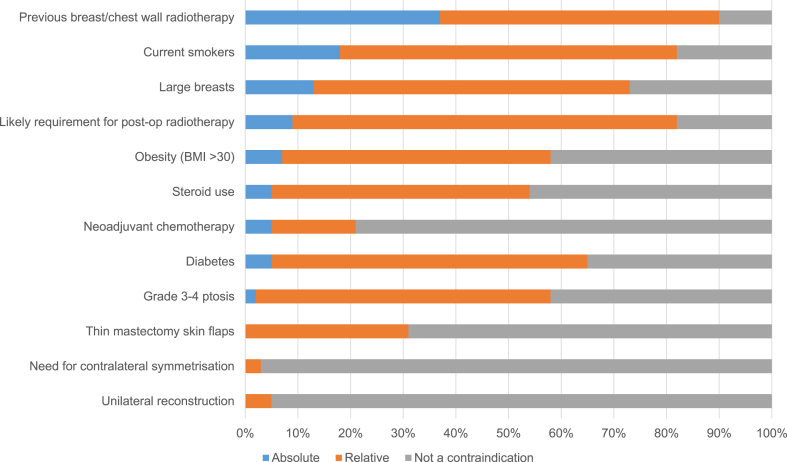
Patient selection for biological mesh-assisted procedures (N = 62).

**Fig. 4:**
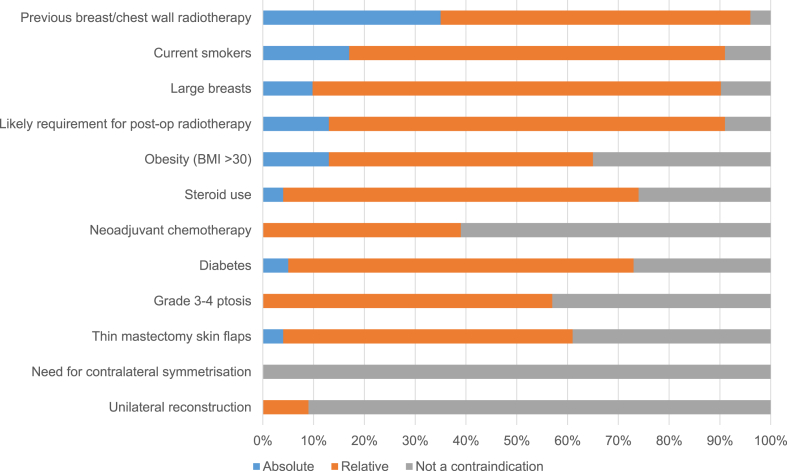
Patient selection for synthetic mesh-assisted procedures (N = 25).

Similar lack of consensus was demonstrated regarding the selection criteria for biological and synthetic mesh-assisted techniques. Previous radiotherapy to the breast, smoking and large breasts were the most commonly reported absolute contraindications for biological meshes, but significant numbers of units reported offering this technique to women in these situations either routinely or on a case-by-case basis (Fig. 3). Patient selection for synthetic mesh-assisted techniques was similarly inconsistent although previous radiotherapy, smoking, obesity and the likely need for post-mastectomy radiotherapy were the most frequently reported reasons for caution when considering this approach (Fig. 4).

## 4 Discussion

This study has provided a valuable insight into the current practice of IBBR in the UK. The procedure is widely-available and represents a significant proportion (70%) of units' reconstructive workload suggesting that greater volumes of IBBR are now being performed than have been previously reported [1]. Units have sufficient expertise to perform implant-based surgery with a median of 2.5 FTE reconstructive breast surgeons and 2.0 FTE plastic surgeons performing a median of 35 immediate implant-based cases per unit per year. Although dermal-sling techniques were widely-available, only 75% of centres offered biological mesh with approximately 20% requiring exceptional funding and just 25% performed synthetic mesh-assisted procedures. The most frequently-used products were Strattice, SurgiMend and TiLOOP but overall, a wide range of meshes were reported. The majority of respondents reported that their reconstructive practice had changed following the introduction of mesh-assisted techniques. Two-thirds reported performing a higher ratio of implant to autologous reconstructions and 40% described an increase in rate of immediate reconstruction overall which was probably related to the availability of these techniques. Patient selection, was shown to be highly-variable between centres with a lack of consensus regarding absolute and relative contraindications for dermal-sling and mesh-assisted procedures. Further work is needed to explore the impact of patient selection on the outcome of IBBR to determine best practice and improve outcomes for patients.

This survey suggests that the availability and practice of IBBR in the UK is evolving and that the introduction of mesh-assisted techniques have resulted in more implant-based surgery being performed. Participating units estimated that 70% of their immediate reconstructions were implant-based which is almost twice that reported in the National Mastectomy and Breast Reconstruction Audit [1]. This may reflect the underrepresentation of plastic surgical units in the study, but reinforces the views of study participants that autologous reconstruction rates and in particular latissimus dorsi flap-based reconstruction, have decreased due to the introduction of mesh-assisted procedures, primarily as more women are now considered suitable candidates for IBBR. These findings may also reflect increased willingness to offer the procedure as recent data suggests that reconstruction rates, overall, have increased [29]. Previous surveys have suggested that only 40% of breast surgeons perform reconstructive surgery [30]. This work suggests that this proportion has increased significantly with responding centres reporting a median of 3.0 FTE breast surgeons per unit, 2.5 of whom were willing to offer reconstructive techniques. This may reflect advanced training programmes such as the UK National Oncoplastic Fellowship scheme and subsequent dissemination of the techniques as fellows become trainers. Another explanation is that only centres engaged in reconstruction have participated in the study. This would be more consistent with recent findings that demonstrate that the increase in the rate of breast reconstruction nationally is due to units with already high rates of reconstruction performing more [29].

The lack of consensus regarding patient selection for mesh-assisted IBBR was unanticipated and of concern. The ABS/BAPRAS ADM guidelines outline areas of caution for the use of biological meshes such as in smokers, women with a body mass index of more than 30; large breasts of greater than 600 g and in women who have had previous breast/chest wall radiotherapy [27]. The evidence for these recommendations, however, is limited and reported variations in practice may reflect this. It is also unclear whether these guidelines can be extrapolated to other non-dermal biological products or to synthetic meshes such as TiLOOP as there is very little published data to support practice [31], [32], [33], [34]. Smoking and obesity, however, have been shown to increase rates of implant loss in traditional two-stage reconstruction [35] and there is no apparent reason why biological or synthetic mesh may influence this. Radiotherapy, before or after implant reconstruction has also been shown to lead to high rates of capsular contracture and decreased patient satisfaction [36]. Recent data to suggest that ADM may offer protection against the adverse effects of radiotherapy [37], [38] may explain why the majority of centres do not consider this to be a contraindication to mesh-assisted IBBR. Data to support the proposed benefits, however are limited [39]. Furthermore, it is unclear if and how potential risks are discussed with patients making decisions about breast reconstruction and why surgeons differ so markedly in which patients they would consider suitable for different techniques. It is possible that more experienced surgeons feel more confident offering IBBR to high risk patients such as smokers or those with a high BMI but further research is needed. Well-designed prospective studies and ideally trials are required to determine best practice and allow predictors for complications to be explored.

This study has limitations. Firstly, it is a practice survey and actual practice may differ significantly from that reported. For example, surgeons may report offering mesh-assisted IBBR to smokers to avoid being considered prejudiced against this group when clinically, they would take this and other risk-factors such as obesity and previous radiotherapy into consideration in their decision-making. In addition, although trainee leads were instructed to complete the questionnaire to reflect the practice of the unit, rather than an individual, it is possible that the responses were skewed by the views of the lead consultant completing the questionnaire. The response rates were relatively low, particularly among plastic surgical units and only 41% of breast units and 23% of plastic surgical units completed the survey. This was a trainee collaborative project so the lack of response may reflect the lack of trainees motivated to engage in the study, a lack of awareness of the project and also the novelty of the methodology in breast and plastic surgical studies. The lower engagement of plastic surgical units, however, is likely to reflect the fact that in the UK, plastic surgeons do not perform significant amounts of immediate IBBR as sufficient numbers of breast surgeons are now prepared to offer the technique without the need for referral. The low response rate, however does raise the issue of response bias as non-participating units may not offer IBBR or may practice in a different way. Recent work has suggested that reconstruction rates vary significantly by cancer network and that although rates of immediate breast reconstruction have increased over time, these have increased predominantly in the centres that were already performing larger volumes of reconstructive surgery rather than those who did not offer that approach [29]. The aim of this survey, however was to determine the numbers of surgeons that were performing mesh-assisted IBBR; the volumes of cases performed; the techniques used and the selection criteria for different techniques to inform the design of a trial. It also aimed to engage centres in the need for evaluation as a preliminary step to trial participation. The data suggest the survey has mainly been completed by high-volume centres and those with on-site plastic surgical expertise who would form the ideal network to participate in future research studies. Finally, this is a rapidly-evolving area with new mesh products being launched and new techniques such as prepectoral reconstruction [40] starting to develop and any future trial must take this into account. Despite these limitations, the survey has demonstrated significant variation in practice among centres performing immediate IBBR surgery in the UK.

High-quality evidence is therefore urgently needed to inform the practice of IBBR and to improve outcomes for patients. This survey is part of iBRA, a prospective multicentre study to inform the feasibility, design and conduct of an RCT comparing new approaches to IBBR [25]. iBRA has recruited over 2000 patients from 75 centres and as such is the largest prospective multicentre study of new approaches of IBBR worldwide. The iBRA study will provide an important resource for hypothesis generation and allow predictors of complications such as implant loss to be explored and best practice established.

Future study designs will be informed by the iBRA data, but if this suggests equivalence between mesh-assisted techniques, an RCT comparing biological and synthetic products may be valuable given the significant cost differential (£1800 vs £200) between the products. A small Austrian RCT comparing TiLOOP and the ADM, Protexa, suggests that this design may be acceptable [19], [20], but as only 25% of units currently offer synthetic mesh-assisted procedures in the UK, more units would need to adopt the technique for such a trial to be feasible in this setting. Other potential study designs may include pre-pectoral vs subpectoral implant placement as products such as BRAXON are gaining in popularity and there may be benefits to a muscle-sparing approach [40]. Outcomes of such approaches are lacking and preliminary safety data is needed before a trial could be considered. Further feasibility work is therefore required to build on the iBRA data to establish the optimal design, comparators and outcome selection for a future trial.

## 5 Conclusions

The iBRA-NPQ has provided a valuable insight into the current practice of IBBR in the UK. It has demonstrated that the uptake and use of implant-based procedures has increased; summarised selection criteria for different techniques and shown that biological, rather than synthetic meshes are predominantly used. Practice, however, is highly variable and reasons for these variations are unclear. There is therefore a need for high-quality evidence to determine best practice and help patients and surgeons make more informed decisions about surgery. This survey is an important first step in developing a well-designed RCT to address the current uncertainties in IBBR and as such is represents a significant contribution to establishing evidence-based reconstructive practice in this rapidly-evolving field.

## Author contributions

SM analysed the data and wrote the first draft of the paper; EC designed the study provided methodological and statistical expertise and analysed the data; PW designed the study provided methodological and statistical expertise for the study; MG inputted on the study design and provided plastic surgical and collaborative expertise and leadership; AJ provided plastic surgical expertise and leadership; LW designed the study and provided trainee collaborative and surgical expertise; JS designed the study and the questionnaire and provided surgical expertise; NB designed the study, refined the questionnaire based on pilot experience and provided surgical expertise; RC designed the study, assisted with data interpretation and provided surgical and methodological expertise; ST designed the study and questionnaire and provided surgical expertise; JB provided methodological advice and support, SP designed the study and questionnaire, wrote the initial proposal, provided trainee collaborative expertise and critically revised the manuscript; CH designed the study, developed the protocol and provided surgical expertise and leadership. SP and CH are joint senior authors on the paper All authors read and approved the final manuscript.

## Collaborators

**The iBRA Steering Group (in alphabetical order) comprises:** N L P Barnes, J M Blazeby, O A Branford, E J Conroy, R I Cutress, M D Gardiner, C Holcombe, A Jain, K McEvoy, N Mills, S Mylvaganam, S Potter, J M Skillman, E M Teasdale, S Thrush, L J Whisker, P R Williamson.

**Local investigators (alphabetically by centre) of the Breast Reconstruction Research Collaborative were:** L Tang, D Nguyen (Abertawe Bro Morgannwg University Health Board NHS Trust); R Johnson, V Muralikrishnan, S Chopra (ABM University Health Board); A Reid, S Benyon (Addenbrookes), C Murphy (Airedale NHS Foundation Trust); F Soliman, V Lefemine (Aneurin Bevan Health Board); S Saha, K Ogedegbe (Barking Havering and Redbridge NHS Trust); O S Olyinka, J R Dicks (Barnsley District General Hospital); N Manoloudakis, F Conroy (Bedford Hospital/Bedfordshire NHS Trust); G Irwin, S McIntosh (Belfast Health and Social Care Trust); I Michalakis (Blackpool Teaching Hospitals NHS Foundation Trust); S Hignett, R Linforth (Bradford Teaching Hospitals NHS Foundation Trust); R Rathinaezhil, H Osman (Brighton and Sussex University Hospitals NHS Trust); K Anesti, M Griffiths, R Jacklin (Broomfield Hospital Mid Essex NHS Trusts); A Waterworth (Calderdale and Huddersfield NHS Trust); R Foulkes, E Davies (Cardiff and Vale); K Bisarya, A Allan, J Leon-Villapalos (Chelsea and Westminster Hospital NHS Foundation Trust); F A K Mazari, I Azmy (Chesterfield Royal Hospital NHS Foundation Trust); S George, F S Fahmy, A Hargreaves, J Seward, S Hignett (Countess of Chester Hospital NHS Foundation Trust); J Henton, T Collin (County Durham and Darlington NHS Foundation Trust); G Irwin, P Mallon (Craigavon Hospital – Southern Health and Social Care Trust); J Turner, W Sarakbi (Croydon University Hospital); I Athanasiou, C Rogers (Doncaster and Bassetlaw Hospitals); M Youssef, T Graja (Dorset County Hospital NHS Foundation Trust); S Huf, H Deol (East and North Herts NHS Trust); R Brindle, S Gawne (East Lancashire Hospitals Trust); D Egbeare (Frimley Health NHS Foundation Trust (Frimley Park Hospital site); I Dash, M Galea (Great Western Hospital - Swindon); S Laws (Hampshire Hospitals NHS Foundation Trust); S Tayeh, L Parvanta (Homerton University Hospital); S Down (James Paget University Hospital); D Westbroek, JW Roberts (Kings College Hospital); J Massey, P Turton, R Achuthan (Leeds Teaching Hospital NHS Trust); M Fawzy, M Dickson (Lister Hospital, East and North Herts); AR Carmichael (London Breast Institute); A Akingboye, R James, K Kirkpatrick (Luton and Dunstable University Hospital); E Nael, R Vidya (Mid Staffordshire NHS Foundation Trust); S Potter, A Thorne (Musgrove Park Taunton); M Rostom, I Depasquale (NHS Grampian); S J Cawthorn (North Bristol NHS Trust); T Gangamihardja (North Middlesex University Hospital); S Joglekar, J Smith (Northern Lincolnshire and Goole Hospitals NHS Foundation Trust); A Halka, D MacMillan (Nottingham University Hospitals NHS Trust); S Clark (Poole Hospital NHS Foundation Trust); B Pearce, L Mansfield (Portsmouth Hospitals NHS Trust, Queen Alexandra Hospital); I King, A Hazari (Queen Victoria Hospitals NHS Foundation Trust, East Grinstead); B Smith (Royal Berkshire Hospital); A J Volleamere (Royal Bolton Foundation Trust); D Egbeare, D Ferguson (Royal Devon and Exeter NHS Foundation Trust); N Barnes C Holcombe (Royal Liverpool and Broadgreen); A Knight, F MacNeill (Royal Marsden NHS Foundation Trust); A Conway, T Irvine (Royal Surrey County Hospital NHS Foundation Trust); S Mylavaganam (Royal Wolverhampton Hospitals NHS Trust (New Cross Hospital); N Dunne, S Kohlhardt (Sheffield Teaching Hospitals); C Hoo, S Kirk (South Eastern Trust, Northern Ireland); J Hu, S Ledwidge (St Bartholomew's Hospital); S Tang, D Banerjee (St George's Healthcare NHS Trust); S Waheed (Surrey and Sussex NHS Trust); V Voynov, S Soumian (University Hospitals North Staffordshire NHS Trust); J Henderson, J Harvey (University Hospital South Manchester); S Robertson, R I Cutress (University Hospital Southampton); S Mylvaganam, R Waters (University Hospitals Birmingham); A Carbone, J Skillman (University Hospitals Coventry and Warwickshire); Ansar Farooq (Warrington & Halton Hospitals NHS Foundation Trust); H Tafazal, D Clarke (Warwick Hospital); D Cocker, L M Lai (West Herfordshire Hospitals NHS Trust); J Winter Beatty, M Barkeji (West Middlesex University Hospital); R Vinayagam (Wirral University Teaching Hospital NHS Foundation Trust); K McEvoy, M Mullan (Worcestershire Acute Hospitals NHS Trust); C Osborne (Yeovil District General Hospital); E Baker, J Piper (York Hospitals NHS Foundation Trust).

## Conflicts of interest

The authors have no conflicts of interest to declare.

## Ethical approval

No ethical approval was required for this study.

## Funding

The iBRA study is funded by the National Institute for Health Research, Research for Patient Benefit Programme (RfPB PB-PG-0214-33065) and pump-priming awards from the Association of Breast Surgery (ABS) and the British Association of Plastic Reconstructive and Aesthetic Surgeons (BAPRAS).

No sponsor was required for this part of the study.

## Disclaimer

This paper presents independent research funded by the National Institute for Health Research (NIHR). The views expressed are those of the author(s) and not necessarily those of the NHS, the NIHR or the Department of Health.
