# Supplementary material for: Variation in the provision and practice of implant-based breast reconstruction in the UK: Results from the iBRA national practice questionnaire
Source: Breast. 2017 Oct;35:182–90. doi: 10.1016/j.breast.2017.07.016 (PMC5590633; doi:10.1016/j.breast.2017.07.016)
Supplement: Supplementary file 1 [file mmc1.pdf]

iBRA

Implant Breast Reconstruction Audit

National Practice Questionnaire

Many thanks for participating in the iBRA study.

We would be very grateful if you could complete the attached questionnaire with the support of the consultants (ideally the lead consultant) in your unit.

This will help us obtain a clear picture of current practice relating to implant-based breast reconstruction and allow us to plan the next stage of the iBRA study.

If you have any questions, please contact us at ibrastudy@gmail.com

SPARCS

THE MAMMARY FOLD
THE NATIONAL BREAST TRAINEES GROUP

RSTN
Reconstructive Surgery Trials Network

ABS
ASSOCIATION OF BREAST SURGERY

RCS
BRISTOL SURGICAL TRIALS CENTRE

BAPRAS
British Association of Plastic Reconstructive and Aesthetic Surgeons

Section 1 – Unit Details

1.1 Name of Trust in which your Unit based

....

1.2 What types of breast reconstruction are offered in your Unit (please tick all that apply)?

None – we refer all patients elsewhere for breast reconstruction	☐
Implant-based reconstruction	☐
Latissimus dorsi flap reconstruction	☐
Pedicled TRAM flap reconstruction	☐
DIEP flap reconstruction	☐
Any other autologous procedures e.g. SGAP, IGAP, SIEA flaps	☐
Therapeutic mammoplasty	☐
Revisional surgery	☐

1.3 In your Unit how many full time equivalent (FTE) of the following specialists with an interest in breast surgery do you have (e.g 2 full time and 1 half time would be 2.5 FTE)?

Consultant Breast Surgeons	....
Non-Consultant Career Grade Breast Surgeons	…………………..
Consultant Plastic Surgeons	....
Non-Consultant Career Grade Plastic Surgeons	....
Breast Care Nurses	....
Breast Reconstruction Nurse Specialists	....
Psychologists	....

1.4 How many of your surgeons perform reconstructive breast surgery?

Consultant Breast Surgeons	....
Non-Consultant Career Grade Breast Surgeons	…………………..
Consultant Plastic Surgeons	....
Non-Consultant Career Grade Plastic Surgeons	....

If breast reconstruction is not offered locally, please tick this box ☐ and skip to section 7.

If your Unit does offer breast reconstruction, please complete Section 2.

Section 2 – Practice of implant-based breast reconstruction in your Unit

2.1 Approximately how many immediate implant-based breast reconstructions are performed in your Unit each year?

……………………………………………………………………………………………………..

2.2 Approximately what percentage of your immediate breast reconstructions are implant-based?

……………………………………………………………………………………………………….

2.3 Do you think the ratio of immediate implant-based to autologous (tissue-based) reconstructions has changed since the introduction of Strattice and other sling procedures?

Yes ☐ No ☐ Unsure ☐

Please provide details for your answer (optional)......................………........................

……………………………………………………………………………………………………….

2.4 Do you think you perform more immediate breast reconstructions since the introduction of Strattice and other sling procedures?

Yes ☐ No ☐ Unsure ☐

Please provide details for your answer (optional)...........……………………………………

……………………………………………………………………………………………………….

2.5 Which approaches to immediate implant-based breast reconstruction are offered in your unit?

	Yes	No	Number of cases performed per year
Standard submuscular placement (two stage procedure)	☐	☐	....
Reduction pattern mastectomy with dermal sling	☐	☐	....
Acellular dermal matrix-assisted reconstruction (e.g Strattice)	☐	☐	....
Other non-dermal biological-assisted reconstruction (e.g.Veritas)	☐	☐	....
TiLOOP-assisted reconstruction	☐	☐	....
Other synthetic-assisted reconstruction	☐	☐	....
Other technique – please state	☐	☐	
....			....

2.6 Which of the following factors are considered absolute or relative contraindications for various implant-based reconstructive techniques in your Unit? Please complete the table using the following codes

A=absolute contraindication
R=relative contraindication
N=not a contraindication.

Please use NA (not applicable) if the technique is not offered in your Unit.

	**Dermal slings**	**Biological mesh e.g. Strattice**	**Synthetic mesh e.g. TiLOOP**
Current smokers			
Large breasts (greater than DD; estimated mastectomy weight >600g)			
Grade 3-4 ptosis			
Obesity (BMI >30)			
Diabetes			
Previous chest wall or breast radiotherapy			
Likely requirement for post-operative radiotherapy			
Unilateral reconstruction			
Need for contralateral symmetrisation			
Thin mastectomy skin flaps			
Neoadjuvant chemotherapy			
Steroid use			
Other (please state)			
Other (please state)			
Other (please state)			

2.7 Do you routinely screen all patients undergoing implant-based reconstruction for the following organisms prior to admission for surgery?

	Yes	No
Methicillin resistant Staph aureus (MRSA)	☐	☐
Methicillin sensitive Staph aureus (MSSA)	☐	☐

2.8 What proportion of your implant-based breast reconstruction cases are performed in a theatre with a laminar flow system?

All cases ☐ Approximately 75% ☐

Approximately 50% ☐ Approximately 25% ☐

None ☐

2.9 What skin prep is routinely used in your Unit for patients undergoing implant-based breast reconstruction?

Aqueous Iodine ☐ Alcoholic iodine ☐

Chlorhexidine ☐ 2% Chlorprep ☐

Surgeon dependent ☐

2.10 Following mastectomy, is the cavity routinely irrigated with saline, water or other prior to inserting the implant?

Yes ☐ No ☐ Surgeon-dependent ☐

2.11 Do the surgeons in your Unit routinely change their gloves/double glove or equivalent prior to handling the implant?

Yes ☐ No ☐ Surgeon-dependent ☐

2.12 Are the following outcomes of different methods of implant-based reconstruction routinely audited in your Unit? Please complete the table for the different techniques using the code

P – Prospectively audited
R – Retrospectively audited
N – Not audited
NA – for procedures not offered in your Unit

	**Subpectoral implants**	**Dermal slings**	**Biological meshes (e.g Strattice)**	**Synthetic meshes (e.g. TiLOOP)**
Short-term complications (< 3 months)				
Long-term complications (>3 months)				
Cosmetic outcomes using pre and post-operative photographs				
Patient-reported outcomes (PROMS)				

Section 3 – Practice of Dermal Sling Procedures

The following questions relate to the practice of <u>dermal slings</u>.

If <u>dermal slings</u> are not performed in your unit, please tick this box ☐ and skip to <u>Section 4</u>.

3.1 How long have dermal slings been offered to patients as an option for immediate breast reconstruction in your unit?

....months/years (please delete as appropriate)

3.2 Which patients are considered good candidates for dermal slings in your Unit? Please list.

....

……………………………………………………………………………………………………..

………………………………………………………………………………………………………

……………………………………………………………………………………………………..

……………………………………………………………………………………………………..

....

3.3 Is there specific written information available to women considering dermal sling reconstruction in your Unit?

Yes ☐ No ☐ Unsure ☐

3.4 If written information is available, are you willing and have permission to provide a copy?

Yes ☐ No ☐ Not applicable ☐

Please attach a copy of your Patient Information Sheet

3.5 What procedure code do you use for dermal sling procedures in your Unit?

....

Continued overleaf

Section 4 – Practice of biological mesh-assisted procedures

The following questions relate to the use of <u>biological meshes</u> such as acellular dermal matrix (ADM).

4.1 Are biological meshes e.g. acellular dermal matrix funded for use in breast reconstruction in your Unit?

Yes, routinely ☐

On exceptional funding application basis ☐

No ☐

If biological meshes are not used in your unit, please tick this box ☐ and skip to <u>Section 5</u>

4.2 How long has ADM/biological mesh-assisted implant-based breast reconstruction been offered to patients considering immediate breast reconstruction in your unit?

.... months/years (please delete as appropriate)

4.3 Which of the following biological meshes are used in your Unit?

Strattice ☐ Permicol ☐

SurgiMend ☐ Protexa ☐

Veritas ☐ BioDesign ☐

Serica ☐

Other...

The following questions refer to the Joint ABS/BAPRAS Guidelines concerning the requirements for Units offering on ADM-assisted breast reconstruction

4.4 Was approval from the New Procedure Committee/Clinical Governance Board obtained before introducing ADM/biological mesh in your Unit?

Yes ☐ No ☐ Unsure ☐

4.5 Is there a formal written protocol or agreed guidelines for the management of patients undergoing ADM/biological mesh assisted reconstruction e.g. regarding antibiotic prophylaxis and drain management in your Unit?

Yes ☐ No ☐ Unsure ☐

4.6 Is there specific written information available to women considering ADM/biological mesh assisted reconstruction in your Unit?

Yes ☐ No ☐ Unsure ☐

4.7 If written information or guidelines are available, are you willing and have permission to provide a copy?

Yes ☐ No ☐ Not applicable ☐

The following questions will allow us to assess variation in the practice and management of ADM/biological mesh-assisted breast reconstruction

4.8 What procedure code do you use for ADM assisted reconstruction in your Unit?

....

4.9 What antibiotic regimen in used in your Unit for ADM-assisted reconstructions?

Antibiotic(s) used including route (iv/oral) and dose

....

Duration of course (days)

....

4.10 What is the policy relating to the use of drains used in ADM/biological mesh assisted reconstruction in your Unit?

Number of drains used..………………………………………………………………………..

Duration drains left in situ (days)..

Or until drain volume less than...mls in 24 hours

Other policy (e.g 2 consecutive days drainage less than 50mls.).......................................

....

4.11 How regularly are patients receiving ADM/biological mesh assisted reconstructions followed up in the early post-operative period?

Frequency (e.g. weekly)...

Duration (e.g. for 4 weeks)...

4.12 How are seromas managed in your Unit?

....

....

Section 5 – Practice of synthetic mesh-assisted procedures

The following questions relate the use of synthetic meshes such as TiLOOP.

If these meshes are not used in your unit, please tick this box ☐ and skip to <u>Section 6</u>

5.1 How long have synthetic mesh-assisted implant-based breast reconstruction been offered as an option to women considering breast reconstruction in your unit?

....

5.2 What synthetic meshes are being used in your Unit?

TiLOOP ☐

Other (please state)...

5.3 Was approval from the New Procedure Committee/Clinical Governance Board obtained before introducing TiLOOP/synthetic mesh in your Unit?

Yes ☐ No ☐ Unsure ☐

5.4 Is there a formal written protocol or agreed guidelines for the management of patients undergoing TiLOOP/synthetic mesh assisted reconstruction e.g. regarding antibiotic prophylaxis and drain management in your Unit?

Yes ☐ No ☐ Unsure ☐

5.5 Is there specific written information available to women considering TiLOOP/synthetic mesh assisted reconstruction in your Unit?

Yes ☐ No ☐ Unsure ☐

5.6 If written information or guidelines are available, are you willing and have permission to provide a copy?

Yes ☐ No ☐ Not applicable ☐

The following questions will allow us to assess variation in the practice and management of TiLOOP/synthetic mesh-assisted breast reconstruction

5.7 What procedure code do you use for synthetic mesh/TiLOOP assisted implant-based breast reconstruction in your Unit?

....

5.8 What antibiotic regimen in used in your Unit for TiLOOP/synthetic mesh-assisted reconstructions?

Antibiotic(s) used including dose and route

....

Duration of course

....

5.9 What is the policy relating to the use of drains used in TiLOOP/synthetic mesh assisted reconstruction in your Unit?

Number of drains used..…………………………………………………………………..

Duration drains left in situ (days)..

Or until drain volume less than...mls in 24 hours

Frequency (e.g. weekly)...

Duration (e.g. for 4 weeks)...

5.11 How are seromas managed following synthetic mesh reconstruction in your Unit?

....

....

Section 6 – Information to determine the feasibility of data collection in the prospective audit

The following questions will help us to determine whether the data collection strategies we are considering for use in the audit will be feasible.

We are planning to collect complication data at 30 days following surgery.

6.1 At what time points post immediate implant-based breast reconstruction do you routinely follow up patients in your Unit?

....

....

We are considering using real-time electronic data collection in the audit to increase the accuracy of the data, avoid the use of paper and save time. The following questions will help us determine the optimal strategy for this.

6.2 Do you have easy and reliable access to computers and the internet in both clinics and theatre in your Unit?

In clinic ☐ In theatre ☐ Neither ☐

6.3 Does your Unit have WiFi access in clinics and theatres?

In clinic ☐ In theatre ☐ Neither ☐

One of the electronic data collection options we are considering involves the use of an iPad 'app'. The app is quick and easy to use; would not require an internet connection to enter data (only to upload) and has been used successfully in South Manchester to collect data on breast reconstruction.

6.4 Would the trainees in your Unit have access to an iPad (either personal or departmental) to use for data collection in the audit?

Personal ☐ Departmental ☐ Neither ☐

The app could be modified for use on an iPhone if there was a sufficient demand for this.

6.5 Would trainees in your Unit have access to an iPhone to use for data collection?

Yes ☐ No iPhone access ☐

There may be a small cost associated with the use of an 'app' for either iPad or iPhone.

6.6 Would your unit be prepared to fund the use of the app for data collection in the audit?

Yes ☐ No ☐ Cost-dependent ☐

6.7 If a cost was involved, what is the <u>maximum</u> level of cost you consider acceptable for this to be funded <u>by your unit</u>?

Less than £1 per month ☐ Up to £5 per month ☐

£5-£10 per month ☐ £10-£15 per month ☐

£15-£20 per month ☐ Would not be prepared to pay ☐

6.8 Would trainees in your Unit be prepared to individually fund the app to facilitate data collection in the audit?

Yes ☐ No ☐ Cost-dependent ☐

6.9 If a cost was involved, what is the <u>maximum</u> level of cost you consider acceptable for this to be funded <u>by individual trainees</u>?

Less than £1 per month	☐	Up to £5 per month	☐
£5-£10 per month	☐	£10-£15 per month	☐
£15-£20 per month	☐	Would not be prepared to pay	☐

6.10 Based on the above, please rank the following methods of data collection in terms of your Unit preferences from 1 (most preferred method) to 6 (least preferred method)

- Paper forms to be sent to audit co-ordinator ☐
- Paper forms with data entry onto an Excel spreadsheet by your Unit e-mailed to audit co-ordinator ☐
- Web-based data entry on a PC by your Unit at point of data collection ☐
- Web-based data entry on a PC by your Unit based on data collected on paper forms ☐
- App based data entry on iPad at point of data collection if cost acceptable ☐
- App based data entry on iPhone at point of data collection if cost acceptable ☐

Section 7 – Contact Details

6.1 Details of Trainee Lead at Centre

Name...Grade..

Email address...

6.2 Details of person completing questionnaire (if different from above)

Name...Grade..

Email address...

6.3 Name of Lead Consultant completing questionnaire

....

6.4 Names of other consultants performing implant-based breast reconstruction in your unit

....

Thank you for completing the questionnaire

Please scan the questionnaire and return it to ibrastudy@gmail.com

or return by post to i-BRA Study, Room 3.12, Canynge Hall, 39 Whatley Road, Clifton Bristol, BS8 2PS
